# Functional engineering of human iPSC‐derived parasympathetic neurons enhances responsiveness to gastrointestinal hormones

**DOI:** 10.1002/2211-5463.13741

**Published:** 2023-12-12

**Authors:** Yuka Akagi, Yuzo Takayama, Yuma Nihashi, Azusa Yamashita, Risa Yoshida, Yasuhisa Miyamoto, Yasuyuki S. Kida

**Affiliations:** ^1^ Cellular and Molecular Biotechnology Research Institute National Institute of Advanced Industrial Science and Technology (AIST) Tsukuba Japan; ^2^ Tsukuba Life Science Innovation Program (T‐LSI), School of Comprehensive Human Sciences University of Tsukuba Tsukuba Japan; ^3^ Analytical Science Laboratories, Asahi Quality & Innovations, Ltd. Moriya Japan; ^4^ School of Integrative & Global Majors (SIGMA) University of Tsukuba Tsukuba Japan

**Keywords:** brain–gut axis, gastrointestinal hormone, induced pluripotent stem cells, signal transduction, vagus nerve

## Abstract

Food‐derived biological signals are transmitted to the brain via peripheral nerves through the paracrine activity of gastrointestinal (GI) hormones. The signal transduction circuit of the brain–gut axis has been analyzed in animals; however, species‐related differences and animal welfare concerns necessitate investigation using *in vitro* human experimental models. Here, we focused on the receptors of five GI hormones (CCK, GLP1, GLP2, PYY, and serotonin (5‐HT)), and established human induced pluripotent stem cell (iPSC) lines that functionally expressed each receptor. Compared to the original iPSCs, iPSCs expressing one of the receptors did not show any differences in global mRNA expression, genomic stability, or differentiation capacities of the three germ layers. We induced parasympathetic neurons from these established iPSC lines to assess vagus nerve activity. We generated GI hormone receptor‐expressing neurons (CCKAR, GLP1R, and NPY2R‐neuron) and tested their responsiveness to each ligand using Ca^2+^ imaging and microelectrode array recording. GI hormone receptor‐expressing neurons (GLP2R and HTR3A) were generated directly by gene induction into iPSC‐derived peripheral nerve progenitors. These receptor‐expressing neurons promise to contribute to a better understanding of how the body responds to GI hormones via the brain–gut axis, aid in drug development, and offer an alternative to animal studies.

## Graphical abstract

Gastrointestinal (GI) hormones transmit food‐derived signals to the brain via peripheral nerves. To address the lack of *in vitro* neuronal models responsive to GI hormones, we generated parasympathetic neurons from human induced pluripotent stem cell lines expressing five GI hormone receptors. These neurons have potential to provide *in vitro* models to reduce animal experiments and enhance our understanding of signal transmission.

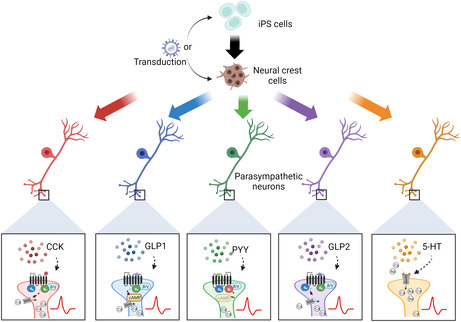

**Abbreviations**
5‐HT: 5‐hydroxytryptamine
BDNF: brain‐derived neurotrophic factor
BMP4: bone morphogenetic protein 4
CCK: cholecystokinin
CCKAR: cholecystokinin A receptor
CHAT: choline acetyltransferase
CHIR: CHIR99021
CNS: central nervous system
DM: dorsomorphin
DMEM: Dulbecco's modified Eagle medium
EB: embryoid body
ESC: embryonic stem cell
FSK: forskolin
GDNF: glial cell‐derived neurotrophic factor
GI: gastrointestinal
GLP1: glucagon‐like peptide 1
GLP1R: glucagon‐like peptide 1 receptor
GLP2: glucagon‐like peptide 2
GLP2R: glucagon‐like peptide 2 receptor
HTR3A: 5‐hydroxytryptamine receptor 3A
IBMX: 3‐isobutyl‐1‐methylxanthine
iPSC: induced pluripotent stem cell
KSR: knockout serum replacement
MEA: microelectrode array
MPC: 2‐methacryloyloxyethyl phosphorylcholine
NCAM1: neural cell adhesion molecule 1
NCC: neural crest cell
ND: neuronal differentiation
NEAA: non‐essential amino acids
NES: nestin
NGFβ: nerve growth factor‐β
NPY2R: neuropeptide Y receptor
NT‐3: neurotrophin‐3
P/S: penicillin–streptomycin
PBS: phosphate‐buffered saline
PEI: polyethyleneimine
PHOX2B: paired like homeobox 2B
PKA: protein kinase A
PLO: poly‐l‐ornithine
PMA: phorbol 12‐myristate 13‐acetate
PRPH: peripherin
PYY: peptide YY
RA: retinoic acid
SB: SB431542
SD: standard deviation
SOX10: SRY‐box transcription factor 10
SOX17: SRY‐box transcription factor 17
TMM: trimmed mean

Gastrointestinal (GI) hormones, predominantly secreted by enteroendocrine cells, are pivotal regulators of physiological responses to food and drugs across various organs, including the brain and intestinal tract. Among these, cholecystokinin (CCK), glucagon‐like peptides 1 and 2 (GLP1 and GLP2), glucose‐dependent insulinotropic peptide, peptide YY (PYY), somatostatin, ghrelin, and serotonin (5‐hydroxytryptamine, 5‐HT), a critical neurotransmitter with diverse roles in the gut–brain axis, are particularly notable. These hormones, through their diverse signaling pathways, play essential roles in regulating appetite, metabolism, and the mobility of GI hormones [1, 2].

The distinct signaling mechanisms of these hormones are as follows:

CCK: On interaction with its ligands, the CCKAR receptor boosts cAMP production through Gs signaling and augments intracellular Ca^2+^ levels via Gq signaling [3].
GLP1: In response to its ligands, GLP1R enhances cAMP production using Gs signaling and increases intracellular Ca^2+^ concentration via the Gq pathway [4, 5].
GLP2: Released from L cells, it activates its receptor, GLP2R, upsurging cAMP production and intracellular Ca^2+^ content through Gs and Gq signaling pathways, respectively [6, 7].
Serotonin (5‐HT): Its receptor, HTR3A, functions as an ionotropic receptor, permitting the influx of Ca^2+^ and Na^+^ ions in response to specific ligands [8].
PYY: Originating from L cells, PYY is crucial in suppressing appetite. It activates the neuropeptide Y receptor (NPY2R) and curtails adenylate cyclase activity through Gi signaling, ultimately inhibiting the cAMP/protein kinase A (PKA) pathway [9].

Systemically, these GI hormones exert their influence on a wide range of organs via circulation, leading to significant modulation of neuronal activities, particularly in the enteric and vagus nerves [1, 10, 11].

With rising ethical concerns over animal use and recognizing inherent species differences that may limit the translation of findings to humans, the demand for alternative research methods in food and drug development has become critical. Human inducing pluripotent stem cells (iPSCs) offers an ethical advantage over animal models and a superior physiological relevance. Human‐derived cells inherently reflect our unique genetic and metabolic intricacies, ensuring a more accurate representation of human biology in experimental outcomes, potentially leading to more relevant therapeutic discoveries. Recent efforts have focused on utilizing *in vitro* approaches to generate human sensory [12, 13, 14, 15], enteric [16, 17, 18], and autonomic nerves [19, 20, 21, 22, 23] from human embryonic stem cells (ESCs) and iPSCs. However, a clear limitation in these efforts is the absence of nerves responsive to GI hormones, which limits our ability to explore the intricate brain–gut axis and understand the etiology and potential interventions for metabolic or neurodegenerative diseases.

In this study, we aimed to generate parasympathetic neurons highly responsive to GI hormones. We induced the expression of key receptor genes (*GLP1R*, *NPY2R*, *CCKAR*, *GLP2R*, and *HTR3A*) in iPSCs, subsequently differentiating them in parasympathetic neurons [21]. Notably, our modified iPSCs, stably expressing GI hormone receptors, demonstrated no karyotypic anomalies and retained robust differentiation potentials. Furthermore, their differentiation into parasympathetic nerves exhibited a pronounced responsiveness to GI hormones.

## Materials and methods

All procedures were performed in accordance with the guidelines of the Committee for the Ethics on Experiments with Human Derivative Samples of the National Institute of Advanced Industrial Science and Technology (AIST) (approval number: 2014‐169). Experiments involving human iPSCs were approved by the Ethics Committee of AIST.

### Cell lines and cell culture

The human iPSC line 201B7 (female) was obtained from the RIKEN Bioresource Center (Tsukuba, Japan). iPSCs were cultured in mTeSR1 cGMP medium (Stemcell Technologies, Vancouver, BC, Canada) in laminin 511‐E8‐coated culture plates (iMatrix511; Nippi, Tokyo, Japan) at 37 °C in an incubator with 5% CO_2_ (Thermo Fisher Scientific, Waltham, MA, USA). The culture medium was replaced daily. At 80–90% confluence, the cell colonies were digested into single cells using accutase (Thermo Fisher Scientific), and the obtained cells were passaged or induced to differentiate into neurons.

HEK293T cells (Lenti‐X 293T Cell line; TaKaRa, Shiga, Japan) were maintained in Dulbecco's Modified Eagle Medium (DMEM; Fujifilm Wako Pure Chemical Industries, Osaka, Japan) supplemented with 10% fetal bovine serum (Biowest, Nuaillé, France), 1% non‐essential amino acids (NEAA) (Fujifilm Wako Pure Chemical Industries), and 1% penicillin–streptomycin (P/S) (Fujifilm Wako Pure Chemical Industries) and cultured in non‐coated culture plates at 37 °C and in an incubator with 5% CO_2_. The culture medium was replaced every 2–3 days. At 80–90% confluence, the cell colonies were digested into single cells using TrypLE Express (Thermo Fisher Scientific). The resulting cells were used for lentiviral transfection and agonist experiments.

### Induction to parasympathetic neurons

Before initiating differentiation, the iPSCs (1–2 × 10^5^ cells·cm^−2^; Cell lines and cell culture section) were transferred to 6‐well plates (Corning, Inc., Corning, NY, USA) coated with 2‐methacryloyloxyethyl phosphorylcholine (MPC) polymer (Lipidure CM5206E; NOF, Tokyo, Japan). The cells were maintained in mTeSR1 cGMP medium containing 10 μm Y‐27632 (Fujifilm Wako Pure Chemical Industries) for 2 or 3 days to form embryoid body (EBs) in a rotary shaker at 95 r.p.m. (OS‐762RC; Optime, Tokyo, Japan). Subsequently, iPSCs were differentiated into neural crest cells (NCCs), as previously reported [21, 23]. Briefly, NCCs were induced from Ebs using knockout serum replacement (KSR) medium and N2 medium with graded addition of nerve growth factors and signaling factors. KSR medium was composed of DMEM‐F12 (Fujifilm Wako Pure Chemical Industries), 20% KSR (Thermo Fisher Scientific), 1% NEAA, 1% monothioglycerol (Fujifilm Wako Pure Chemical Industries), and 1% P/S. N2 medium consisted of DMEM‐F12, 1% N2 supplement (Fujifilm Wako Pure Chemical Industries), 1% NEAA, and 1% P/S. When EB formation was considered day 0 of induction, the medium was changed at days 2, 5, 7, 9, and 12. The following nerve growth factors and inhibitors were used: dorsomorphin (DM; Sigma‐Aldrich, St. Louis, MO, USA), SB431542 (SB; Sigma‐Aldrich), bFGF (Fujifilm Wako Pure Chemical Industries), CHIR99021 (CHIR; Cayman Chemical, Ann Arbor, MI, USA), IWR1 (Sigma‐Aldrich), SANT1 (Sigma‐Aldrich), and human bone morphogenetic protein 4 (BMP4; Fujifilm Wako Pure Chemical). On day 13, the induced NCCs were dissociated with TrypLE Express (Thermo Fisher Scientific), and the cells were plated in culture plates coated with poly‐l‐ornithine (PLO; Sigma‐Aldrich) and laminin (Sigma‐Aldrich) in neuronal differentiation (ND) medium. The ND medium consisted of N2 medium, 10 μm forskolin (FSK), 50 μg·mL^−1^ ascorbic acid, 10 ng·mL^−1^ recombinant human brain‐derived neurotrophic factor (BDNF), 10 ng·mL^−1^ recombinant human glial cell‐derived neurotrophic factor (GDNF), 10 ng·mL^−1^ recombinant human nerve growth factor‐β (NGFβ), 10 ng·mL^−1^ recombinant human neurotrophin 3 (NT‐3), 10 ng·mL^−1^ phorbol 12‐myristate 13‐acetate (PMA) (all from Fujifilm Wako Pure Chemical Industries), and 1 μm retinoic acid (RA) (Sigma‐Aldrich). We plated 2.5–5 × 10^5^ cells·cm^−2^. The spent ND medium was changed twice a week.

### Overexpression of receptor genes via lentivirus transduction

Figure S1 presents the protocol used for this experiment. Lentiviral vectors were acquired from VectorBuilder (VectorBuilder Japan, Inc., Kanagawa, Japan). Detailed information on the vector is given in Table S1. The mCherry expression vector (pLV‐Puro‐EF1A‐mCherry) was used as a control. pMD2.g plasmid (Addgene plasmid# 12259; RRID: Addgene 12259, Watertown, MA, USA) and psPAX2 plasmids (Addgene plasmid# 12260; RRID: Addgene 12260) were used as packaging plasmids. To produce lentiviruses, HEK293T cells were transduced with lentiviral vectors and packaging constructs containing 2 μg of lentivirus plasmids, 1 μg of pMD2.g, and 1 μg of psPAX2 plasmids using polyethyleneimine (PEI; Polysciences Inc., Warrington, PA, USA). After adding the PEI‐containing plasmid solution, the cells were incubated at 37 °C for approximately 16–20 h. Subsequently, the medium was replaced with fresh DMEM containing 10% FBS, 1% P/S, and NEAA. After 48 and 98 h, the culture supernatants were collected and passed through a 0.45‐μm PVDF syringe filter. The viral solutions were concentrated using a Lenti‐X™ Concentrator (TaKaRa) and stored at −80 °C until further use. For transduction, the iPSCs were plated in plates coated with laminin 511‐E8 (iMatrix511; Nippi) in mTeSR1cGMP medium and incubated overnight at 37 °C; the viral supernatant was added the next day. After 12–18 h, the spent medium was replaced with fresh mTeSR1 cGMP medium. Subsequently, the cells were selected using 0.3 μg·mL^−1^ puromycin after 5 days of transduction. Puromycin was added throughout the culturing of transgenic iPSCs and differentiated nerves. Only the cells resistant to puromycin due to gene transfer survived. For gene transduction in differentiated neurons, NCCs induced from wild‐type iPSCs (day 13 of induction) were dissociated using TrypLE Express (Thermo Fisher Scientific), and the cells were plated in culture plates coated with PLO (Sigma‐Aldrich) and laminin (Sigma‐Aldrich) in ND medium (Induction to parasympathetic neurons section). Viral supernatants were added after 7 days of seeding. After 12–18 h, the medium was replaced with fresh ND medium. Cells were selected using 0.3 μg·mL^−1^ puromycin after 5 days of transduction, and only the cells resistant to puromycin due to gene transfer survived.

### Chromosomal G‐band analyses

Chromosome safety analysis of wild‐type and transduced iPSC lines was performed at the Nihon Gene Research Laboratories (Miyagi, Japan). Briefly, Giemsa staining was performed for sub‐confluent cells. Among the stained cells, 20 mitotic cells (metaphase) were randomly selected and evaluated for their G‐banding patterns [24].

### *In vitro* differentiation into three germ layers

Directed differentiation into all three germ layers was achieved using the STEMdiff™ Trilineage Differentiation Kit (StemCell Technologies), according to the manufacturer's instructions. Briefly, after harvesting iPSCs using accutase, they were isolated, counted, and seeded at the recommended cell densities (Ectoderm; 2 × 10^6^ cells, Mesoderm; 5 × 10^5^ cells, Endoderm; 2 × 10^6^ cells) in 6‐well plates coated with laminin 511‐E8 (iMatrix511; Nippi) lineage‐specific medium. The spent medium was changed daily until day 5 for mesoderm and endoderm differentiation and day 7 for ectoderm differentiation. Ectoderm differentiation did not exhibit sufficient increase in the expression of differentiation marker genes (data not shown). Thus, ectoderm differentiation was performed using the neuronal differentiation induction method as previously described [21]. Ectoderm differentiation was confirmed using NCCs on day 13 of induction.

### Immunochemical staining

Immunochemical experiments were performed as previously described [25]. Briefly, the transduced HEK293T cells, iPSC lines, and neurons were fixed in 4% paraformaldehyde (Fujifilm Wako Pure Chemical Industries) for 30 min, permeabilized using 0.1% Triton X‐100 (Fujifilm Wako Pure Chemical Industries) in phosphate‐buffered saline (PBS) for 10 min, and blocked with 4% Block Ace (DS Pharma Biomedical, Osaka, Japan) in 0.01% Triton X‐100 for 1 h at room temperature (approximately 25 °C). The following primary antibodies were used: mouse anti‐microtubule‐associated protein (MAP2; 1 : 1000; ab11267; Abcam, Cambridge, UK), rabbit anti‐MAP2 (1 : 1000; ab32454; Abcam), anti‐GLP1R (1 : 50; PA5‐97789; Thermo Fisher Scientific), anti‐NPY2R (1 : 100; SAB4502029; Sigma‐Aldrich), anti‐CCKAR (1 : 100; ab115287; Abcam), anti‐GLP2R (1 : 50; MAB4285; R&D Systems, Minneapolis, MN, USA), anti‐HTR3A (1 : 250; MA5‐31771; Thermo Fisher Scientific), and Hoechst 33342 (H342; Dojindo Laboratories, Kumamoto, Japan). The following secondary antibodies were used: Alexa Fluor 555 (F[ab])2 goat anti‐rabbit IgG [H + L] secondary antibody (A21430; Thermo Fisher Scientific) and Alexa Fluor 488 (F[ab′]2 goat anti‐mouse IgG [H + L] secondary antibody) (A11017; Thermo Fisher Scientific).

### RNA‐seq

Total RNA was isolated from differentiated cells using a Nucleospin RNA Kit (U0955B; MACHEREY‐NAGEL GmbH & Co. KG, Dueren, Germany). RNA purity and concentration were determined using a NanoDrop Lite spectrophotometer (Thermo Fisher Scientific). RNA‐seq was performed by Macrogen (Seoul, Korea). The library was prepared using > 1 μg of cDNA, and its quality was analyzed using an Agilent 2100 Bioanalyzer (Agilent Technologies Japan, Ltd., Tokyo, Japan). The obtained library was sequenced using a NovaSeq 6000 platform (Illumina, San Diego, CA, USA) to produce paired end reads (150 base pairs). The acquired data were mapped and quantified using star [26] (version 2.7.9a) and rsem [27] (version 1.3.3), with hg38 as the reference genome (gene annotation: Ensembl GRCh38). Subsequently, the read count data were normalized to the TMM using edger [28] (version 3.32.1) in r software (version 4.0.4) to analyze the differentially expressed genes. Hierarchical clustering analysis was performed to determine the similarity of each dataset, as per Spearman's rank correlation coefficient using the Cor function in r.

### cAMP accumulation assay

HEK293T cells were seeded (8 × 10^4^ cells per well) in 96‐well plates coated with 0.1% (w/v) gelatin (Fujifilm Wako Pure Chemical Industries) in DMEM containing 10% FBS, 1% NEAA, and 1% P/S for 24 h. To verify ligand responsiveness of GLP1R‐HEK293T cells, the spent medium was changed with the abovementioned medium supplemented with 0, 0.01, 0.1, 1, 10, and 100 nm GLP1 (4280‐v; Peptide Institute, Inc., Osaka, Japan) and 0.5 mm 3‐Isobutyl‐1‐methylxanthine (IBMX) (I7018 250MG; Sigma‐Aldrich) at a final volume of 100 μL and incubated at 37 °C for 30 min. To evaluate the responsiveness of NPY2R‐HEK293T cells, the spent medium was changed with the abovementioned medium supplemented with 0, 1, 10, and 100 nm PYY (4400‐v; Peptide Institute, Inc.), 0.5 mm IBMX, and 10 μm FSK at a final volume of 100 μL and incubated at 37 °C for 30 min. The supernatant was discarded, and the cells were washed with PBS containing 0.5 mm IBMX. For cell lysis, 0.1 m HCl was added to the cells (100 μL per well), and the plates were incubated at 37 °C for 10 min. After centrifuging the cell lysate at 1000 ***g*** for 10 min, the supernatant was collected and stored at −80 °C until further analyses. cAMP concentration was measured using a cAMP ELISA kit (ADI‐900‐163; Enzo Life Sciences, Farmingdale, NY, USA). The protein concentration of the cell lysate was measured using a Pierce™ BCA Protein Assay Kit (23225; Thermo Fisher Scientific), and the amount of cAMP detected was corrected for the protein concentration in each sample.

### Calcium imaging

Transduced HEK293T and neurons were incubated with 10 μm Fluo‐8 AM, a calcium indicator (AAT Bioquest, Pleasanton, CA, USA), in each culture medium (Cell lines and cell culture and Induction to parasympathetic neurons sections) at 37 °C for 30 min. Subsequently, the ND medium containing Fluo‐8 AM was replaced with Ringer's solution (148 mm NaCl, 2.8 mm KCl, 2 mm CaCl_2_, 1 mm MgCl_2_, 10 mm HEPES, and 10 mm glucose; pH 7.4). The agonists used in this experiment were as follows: CCK‐33 (4201‐s; Peptide Institute, Inc.), CCK‐Octapeptide (26–33) (CCK‐8) (4100‐v; Peptide Institute, Inc.), GLP1 (7–37), GLP2 (4276‐v; Peptide Institute, Inc.), 5‐HT (H9526; Sigma‐Aldrich), and PYY (3–36). The samples were imaged using an inverted microscope (IX71; Olympus, Tokyo, Japan). Fluorescence (excitation: 490 nm, emission: 525 nm) was detected using a confocal spinning disk confocal laser scanning unit (CSU‐W1; Yokogawa Electric, Tokyo, Japan) and an LED light (X‐Cite 120LED; Opto Science, Tokyo, Japan). During fluorescence analysis, 10 μm nicotine (in ethanol; Sigma‐Aldrich) was added to Ringer's solution to verify parasympathetic nerve function. A frame rate of 0.8 s^−1^ was used for imaging. The recorded fluorescence signals were analyzed using imagej software (National Institutes of Health, Bethesda, MD, USA).

### Reverse transcription (RT)‐qPCR

Total RNA was isolated from the induced cells using a Nucleospin RNA Kit (TaKaRa). RNA purity and concentration were determined using a NanoDrop Lite spectrophotometer (Thermo Fisher Scientific). We reverse‐transcribed 1000 ng of total RNA to cDNA using a ReverTra Ace qPCR RT Kit (TOYOBO, Osaka, Japan). qPCR was performed in a LightCycler® 96 System (F. Hoffmann‐La Roche, Ltd., Basel, Switzerland) using the THUNDERBIRD SYBR qPCR Mix (TOYOBO) under the following conditions: 95 °C for 600 s, 60 °C for 10 s and 72 °C for 10 s; 45 cycles of 95 °C for 10 s, 60 °C for 10 s, and 72 °C for 10 s; and melting at 95 °C for 10 s, 65 °C for 60 s, and 97 °C for 1 s. The expression of the genes was normalized to *36B4* expression (housekeeping gene). The primer sequences are listed in Table S2.

### Microelectrode array recording of neuron activity

Induced pluripotent stem cell ‐derived neurons were cultured in PLO/laminin‐coated CytoView microelectrode array (MEA) 6‐well plates with 64 embedded electrodes (M384‐tMEA‐6 W; Axion BioSystems, Inc., Atlanta, GA, USA) or CytoView MEA 24‐well plates with 16 embedded electrodes (M384‐tMEA‐24W; Axion BioSystems, Inc.). Extracellular neuronal activity from the neurons was assessed using an MEA recording system (MAESTRO Edge; Axion BioSystems, Inc.) on day 27 of differentiation. Spontaneous neuronal firing was measured 5 min before and after ligand addition in each well. Responsiveness to ligands was measured in three wells. The ratio (Hz) after and before ligand addition for each electrode was determined; frequency ≥ 0.1 Hz was used as the threshold.

### Quantification and statistical analysis

All data are expressed as mean ± standard deviation (SD) of triplicate measurements. Differences between experimental groups were analyzed using Student's *t*‐test or Welch's *t*‐test (two groups). Differences between more than two groups were analyzed using one‐way ANOVA, and Dunnett's or Williams's *post‐hoc* method was used for multiple comparisons. Differences were considered statistically significant at *P* < 0.05.

## Results

### Response of GI hormone receptors in HEK293T cells

**Fig. 1:**
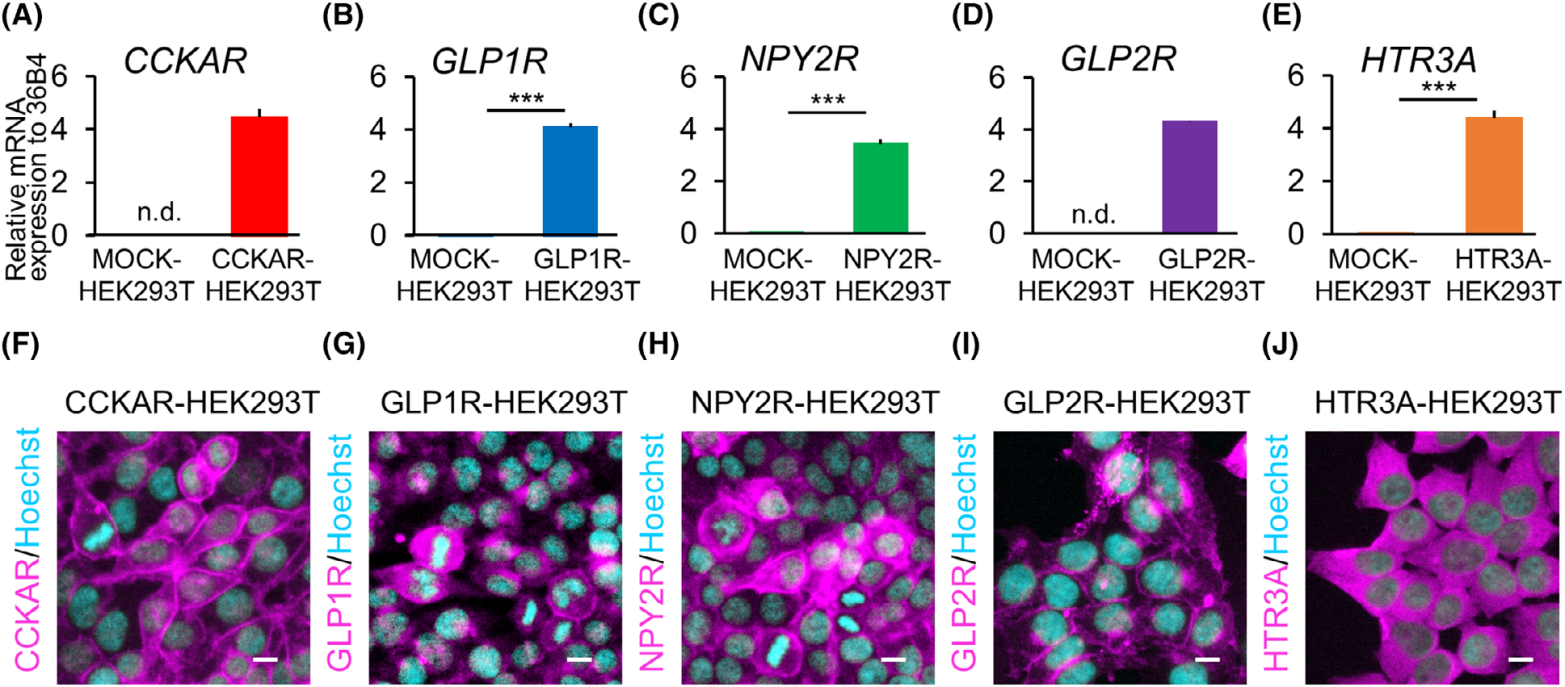
Establishment of HEK293T cells expressing gastrointestinal (GI) hormone receptors. (A–E) mRNA expression of target transgenes (*CCKAR*, *GLP1R*, *NPY2R*, *GLP2R*, and *HTR3A*) in MOCK‐HEK293T cells or GI hormone receptor‐transduced HEK293T cells (CCKAR‐HEK293T, GLP1R‐HEK293T, NPY2R‐HEK293T, GLP2R‐HEK293T, and HTR3A‐HEK293T) (*n* = 3). Error bars represent standard deviation (SD). Student's *t*‐test. ***P < 0.001; n.d., not detected. (F–J) Immunofluorescence staining of GI hormone receptors on HEK293T cells (magenta: GI hormone receptor, cyan: Hoechst 33342). Scale bar: 10 μm.

To verify the response of the induced GI hormone receptors, the receptor genes (*GLP1R*, *NPY2R*, *CCKAR*, *GLP2R*, and *HTR3A*) were transduced using a lentiviral vector in HEK293T cells (Fig. S1). Compared to that in the mCherry‐transduced‐HEK293T cells (MOCK‐HEK293T), mRNA expression of each receptor was significantly increased in the receptor‐transduced HEK293T cells (Fig. 1A–E). Moreover, immunofluorescence staining (Fig. 1F–J) confirmed the expression of the corresponding receptor on the cell membranes of CCKAR‐HEK293T (Fig. 1F), NPY2R‐HEK293T (Fig. 1H), and GLP2R‐HEK293T cells (Fig. 1I). In contrast, GLP1R‐HEK293T and HTR3A‐HEK293T cells exhibited high expression of their receptors not only in the cell membrane but also in the cytoplasm (Fig. 1G,J).

**Fig. 2:**
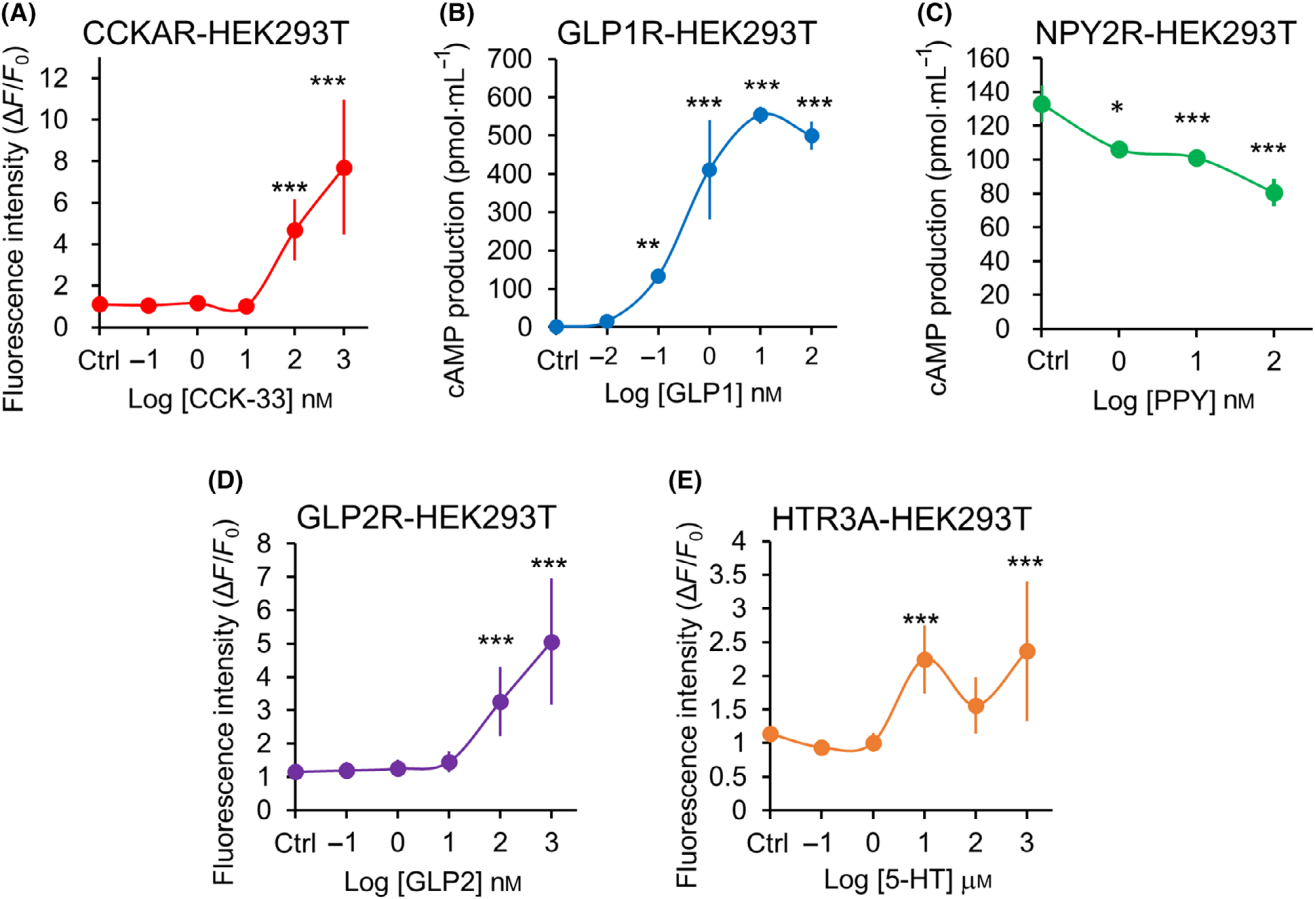
Responsiveness of each receptor on HEK293T cells expressing GI hormone receptors. (A) Concentration‐response curve of intracellular Ca^2+^ concentrations (Δ*F*/*F*_0_) of CCKAR‐HEK293T cells in response to CCK‐33 treatment (*n* = 10; error bars represent SD). (B) Concentration‐response curve of cAMP production (pmol·mL^−1^) by GLP1R‐HEK293T cells in response to GLP‐1 treatment (*n* = 3; error bars represent SD). (C) Concentration‐response curve of cAMP production (pmol·mL^−1^) by NPY2R‐HEK293T cells in response to PYY treatment (*n* = 3; error bars represent SD). (D) Concentration‐response curve of intracellular Ca^2+^ concentrations (Δ*F*/*F*_0_) of GLP2R‐HEK293T cells in response to GLP2 treatment (*n* = 20; error bars represent SD). (E) Concentration‐response curve of intracellular Ca^2+^ concentrations (Δ*F*/*F*_0_) of HTR3A‐HEK293T cells in response to 5‐HT treatment (*n* = 10; error bars represent SD). Data were analyzed using one‐way ANOVA followed by Dunnett's *post‐hoc* test. *P < 0.05, **P < 0.01, ***P < 0.001 vs. 0 nm or 0 μm (Control, Ctrl). (A, D, E) Δ*F*: average fluorescence after ligand addition, *F*_0_: average fluorescence before ligand addition. See also Fig. S2.

We then observed that all receptor proteins expressed by the lentivirus‐encoded transgenes were responsive to their corresponding ligands in HEK293T cells. The response of CCKAR‐HEK293T cells to > 100 nm CCK‐33 (Fig. 2A) was assessed by monitoring the changes in intracellular Ca^2+^ concentration. Similar responses were observed for CCK‐8 (different molecular weights), indicating that > 10 nm CCK‐8 can increase intracellular Ca^2+^ concentration (Fig. S2A). GLP1R‐HEK293T cells showed increased cAMP production with increasing GLP1 concentration (Fig. 2B). While assessing the function of Gi protein in NPY2R, NPY2R‐HEK293T cells showed that intracellular cAMP levels decreased in a concentration‐dependent manner (Fig. 2C). Additionally, as NPY2R is a Gq‐type G protein‐coupled receptor, NPY2R‐HEK293T cells responded to > 10 nm PYY and exhibited increased intracellular Ca^2+^ concentrations (Fig. S2B). The responsiveness of GLP2R‐HEK293T cells was assessed by Ca^2+^ imaging and showed a responsive to > 100 nm GLP2 (Fig. 2D). Finally, HTR3A‐HEK293T cells were responsive to > 10 μm 5‐HT, as determined by measuring their intracellular Ca^2+^ concentration (Fig. 2E).

### Establishment of human iPSC lines expressing GI hormone receptors

**Fig. 3:**
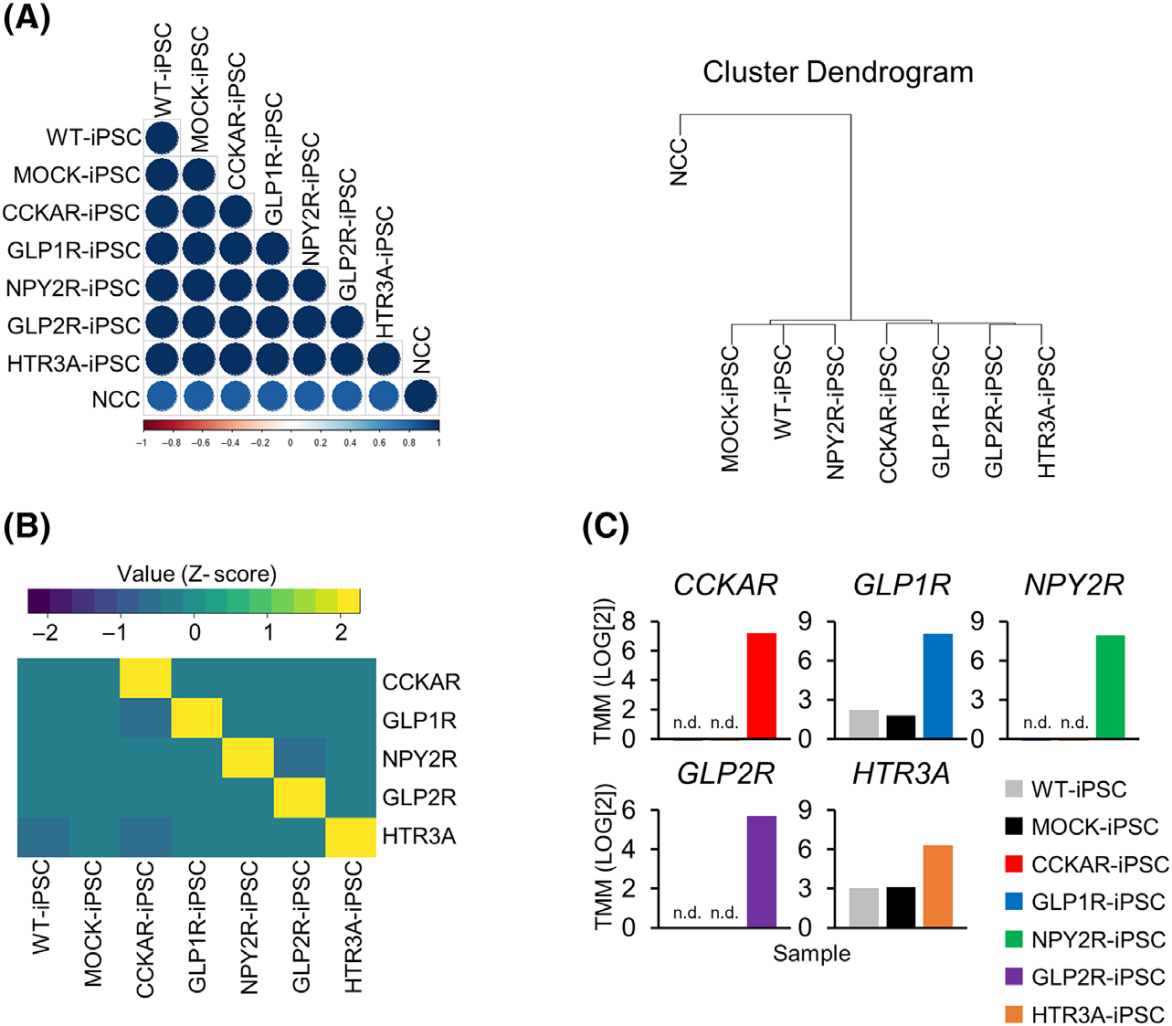
Transcriptomic analysis of GI hormone receptor‐expressing iPSC lines. (A) Heatmaps and clustering dendrograms based on Spearman's rank correlation coefficients of wild‐type iPSCs (WT‐iPSCs), MOCK‐expressing iPSCs (MOCK‐iPSCs), and receptor‐expressing iPSC lines compared to those of NCCs differentiated from WT‐iPSCs (day 13 of induction [21]) as negative control. (B) Heatmap based on the expression of receptor genes on WT‐iPSCs and MOCK‐iPSCs (*z*‐score values were based on the cell counts). (C) Trimmed mean (TMM) values of target receptors on WT‐iPSCs, MOCK‐iPSCs, and transgene‐expressing iPSC lines (n.d., not detected). See also Fig. S3.

Using the lentivirus vectors, we established iPSC lines (CCKAR‐iPSCs, GLP1R‐iPSCs, GLP2R‐iPSCs, HTR3A‐iPSCs, and NPY2R‐iPSCs) that could stably express each receptor. Transcriptomics analyses (RNA‐seq) were conducted to examine the global mRNA expression and receptor overexpression in these cells. A color map, constructed using Spearman's rank correlation coefficients, along with a clustering tree diagram, is presented in Fig. 3A. The correlation coefficients between receptor‐transduced iPSCs and wild‐type iPSCs (WT‐iPSCs) or MOCK‐iPSCs were > 0.98, indicating a high degree of similarity between the iPSC lines. However, the correlation coefficients between differentiated NCCs derived from WT‐iPSCs (day 13 of induction [21, 23]) as a negative control and each type of iPSC line ranged 0.69–0.7, which were lower than the values obtained for different iPSC lines. Clustering analysis revealed that NCCs and iPSC lines clustered separately. The receptor‐transduced iPSC lines exhibited gene expression patterns similar to those of the MOCK‐iPSCs and WT‐iPSCs. Furthermore, each receptor was specifically expressed in the transgenic lines (Fig. 3B). Trimmed mean (TMM) values revealed that the expression of specific receptors was > 8‐fold higher than their endogenous expression in WT‐iPSCs or MOCK‐iPSCs (Fig. 3C).

### Verification of the genomic stability and pluripotency of engineered iPSCs

Chromosome number aneuploidy and structural aberrations in the engineered iPSC lines were evaluated using the G‐band method (Fig. S3A). All iPSC lines, including WT‐iPSCs, displayed a normal karyotype of 46XX. Differentiation abilities toward three germ layers *in vitro* were assessed by verifying the expression of marker genes through quantitative PCR (Fig. S3B–F). On day 13 of neuronal differentiation, the expression of nestin (*NES*), a marker of ectoderm differentiation, increased in all cell lines (Fig. S3B–F). On day 5, mesoderm cells exhibited significantly increased gene expression of neural cell adhesion molecule 1 (*NCAM1*) (Fig. S3B–F). Similarly, an increase in the expression of SRY‐box transcription factor 17 (*SOX17*), involved in endoderm differentiation, was observed in day‐5 endoderm cells (Fig. S3B–F). Thus, the engineered iPSCs exhibited genomic stability and differentiation capability.

### Differentiation of engineered iPSCs into parasympathetic neurons

**Fig. 4:**
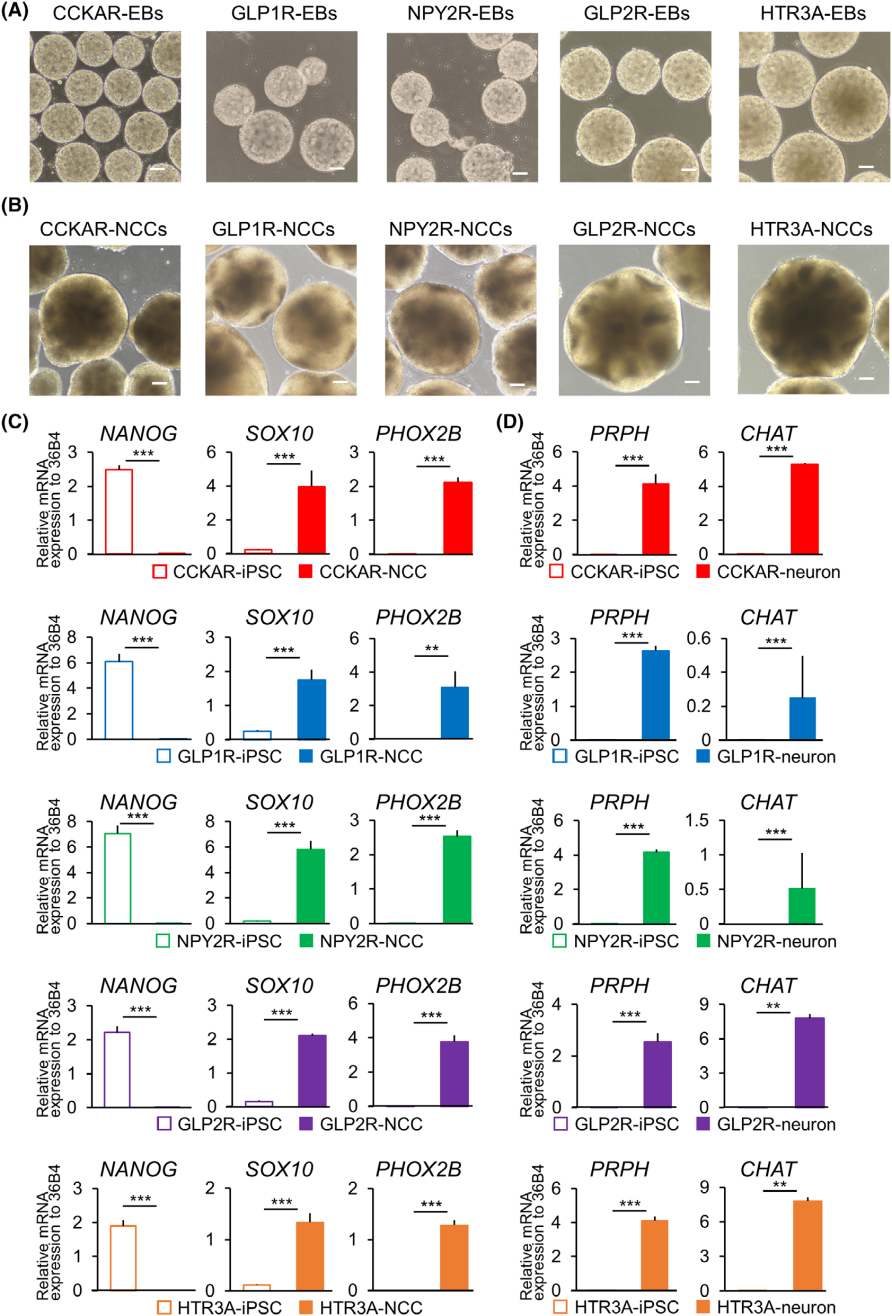
Generation of human parasympathetic neurons expressing GI hormone receptors. (A) Phase‐contrast images of transgene‐expressing EBs (scale bar: 100 μm) and (B) NCCs on day 13 of induction (scale bar: 100 μm). (C) Relative mRNA expression levels of undifferentiated marker genes (*NANOG*) and NCC markers (*SOX10* and *PHOX2B*) compared to that of *36B4* (housekeeping gene) in transgene‐expressing iPSCs and NCCs on day 13 of induction. (D) mRNA expression levels of parasympathetic nerve marker genes (peripherin (*PRPH*) and *CHAT*) compared to that of *36B4* in transgene‐expressing iPSCs and neuron on day 40 of induction. Data were analyzed using Student's *t*‐test. *n* = 3; error bars represent SD; **P < 0.01, ***P < 0.001 vs. iPSCs. See also Fig. S4.

To verify if GI hormones could regulate neuronal function, we differentiated established iPSC lines into parasympathetic neurons, a component of the vagus nerve. Each iPSC line formed EBs (200–400 μm in diameter; day 0 of induction; Fig. 4A), and neuronal spheres were observed with no receptor‐dependent differences in the formation rate nor size (“NCC”; day 13 of induction; Fig. 4B). The presence of NCCs in the neuronal spheres was confirmed with a decrease in the expression of undifferentiated cell marker (*NANOG*) and an increase in the expression of NCC marker (*SOX10* and *PHOX2B*) compared to that in undifferentiated iPSCs (Fig. 4C). The neurons obtained on day 40 after induction exhibited higher expression of parasympathetic nerve markers (peripherin; *PRPH* and choline acetyltransferase; *CHAT*) than undifferentiated iPSCs (Fig. 4D).

**Fig. 5:**
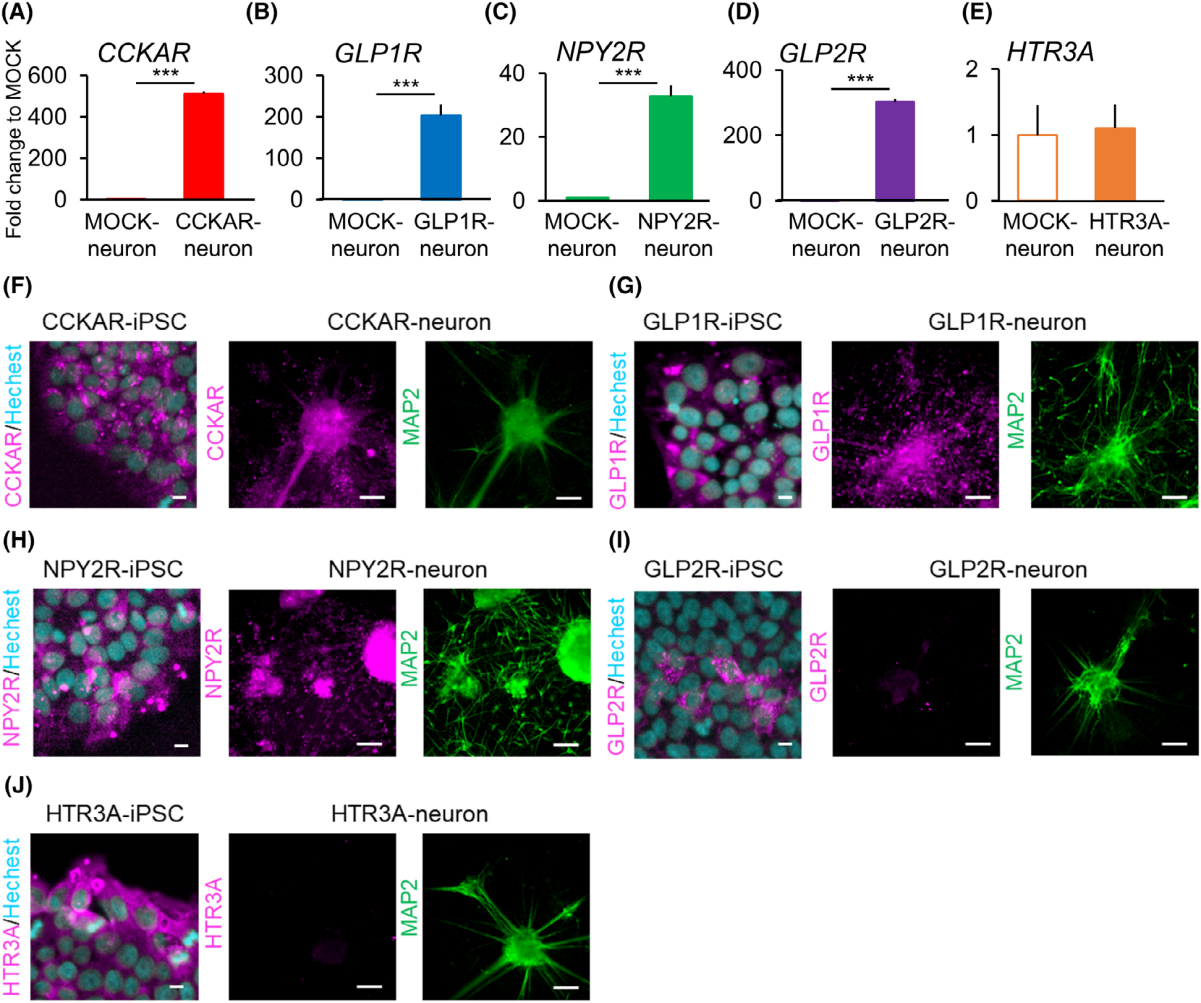
Expression of GI hormone receptors on induced neurons. (A–E) Fold‐changes in the expression of transgenes in MOCK‐expressing neurons (MOCK‐neurons) and transgenic neurons on day 40 of induction. *n* = 3; error bars represent the SD; Student's *t*‐test; ***P < 0.001 vs. MOCK‐neurons. (F–J) Immunofluorescence staining of GI hormone receptors on transgene‐expressing iPSCs and neurons on day 40 of induction (magenta: GI hormone receptor, green: MAP2, cyan: Hoechst 33342). Scale bar in iPSC photographs: 10 μm. Scale bar in neuron photographs: 100 μm.

Higher expression of *CCKAR*, *GLP1R*, *GLP2R*, and *NPY2R* mRNA was detected in the parasympathetic neurons than in MOCK‐iPSC‐derived neurons (MOCK‐neurons) (Fig. 5A–E). In CCKAR‐, GLP1R‐, and NPY2R‐neurons, immunostaining revealed positive expression for each target receptor protein in iPSCs and neurons (Fig. 5F–H). Each receptor protein was expressed in the cytoplasm and neurites. In contrast, immunostaining revealed that *GLP2R* was expressed in a few GLP2R‐iPSCs, with a positivity rate of > 1%. No iPSC lines ubiquitously overexpressing GLP2R protein were established, and overexpression in the corresponding neurons was not observed (Fig. 5I). In contrast, *HTR3A* expression in HTR3A‐iPSC‐derived neurons (HTR3A‐neurons) was similar to that in MOCK‐neurons, and overexpression was not observed; induced *HTR3A* expression was immediately downregulated in post‐differentiated HTR3A‐neurons compared to that in pre‐differentiated HTR3A‐iPSCs (approximately 243‐fold lower expression) (Fig. S4). Additionally, all HTR3A‐iPSCs expressed HTR3A protein; however, *HTR3A* expression was not observed in the neurons after differentiation (Fig. 5J).

### Verification of the response of engineered neurons to GI hormones

**Fig. 6:**
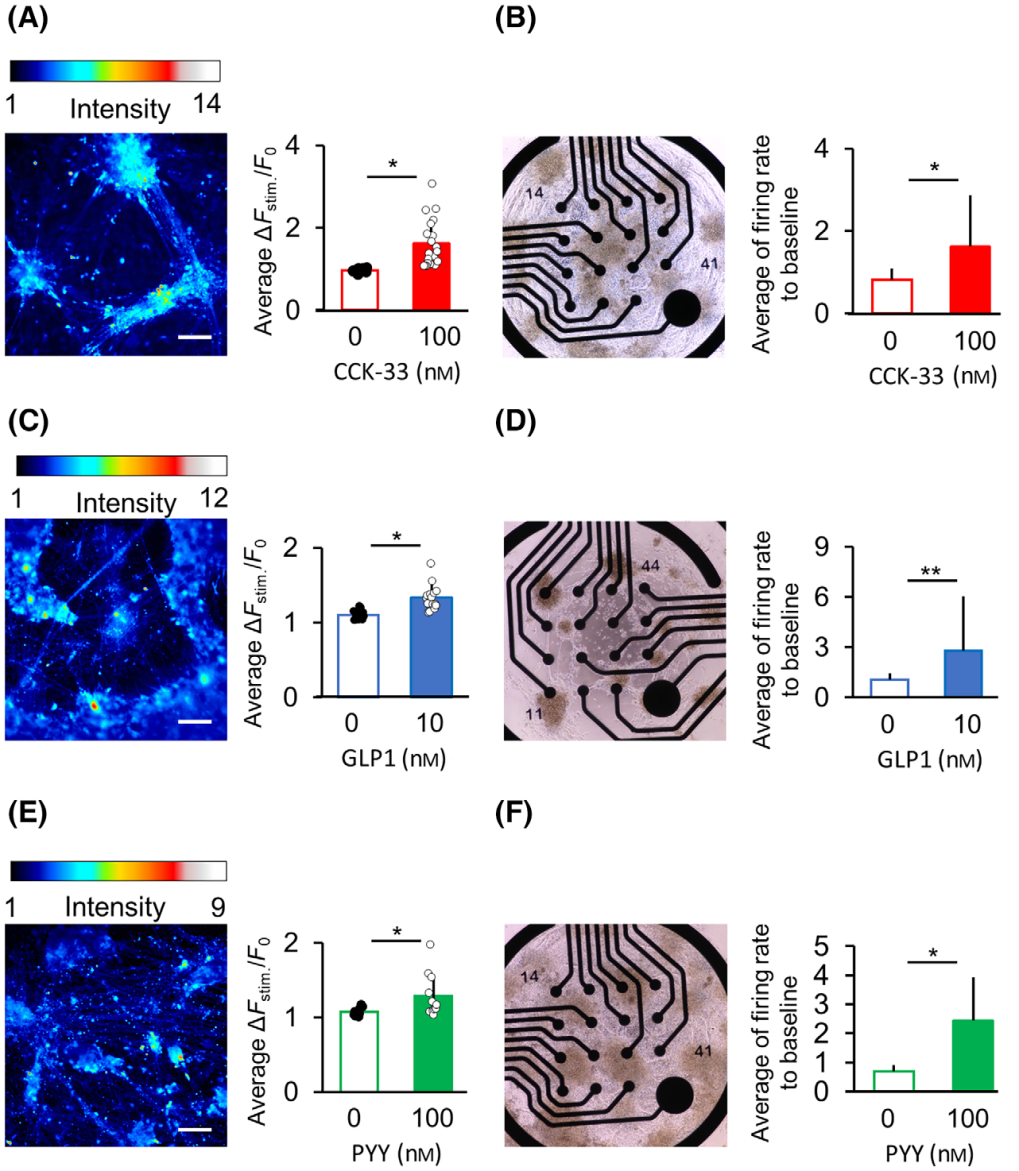
Verification of ligand responsiveness in engineered neurons. (A, B) Responsiveness test to 100 nm CCK for CCKAR‐neurons. (C, D) Responsiveness of GLP1R‐neurons to 10 nm GLP1. (E, F) Responsiveness of NPY2R‐neurons to 10 nm PYY. (A, C, E) Ca^2+^ imaging of engineered neurons on day 35 in response to ligands. Color bars represent fluorescence intensity. Scale bar: 100 μm (left panel). Right panel shows the ratio of change (Δ*F*_stim_/*F*_0_) in the average fluorescence intensity after ligand addition (Δ*F*_stim_) and average fluorescence intensity before ligand addition (*F*_0_). (A) CCKAR‐neurons; *n* = 20, (C) GLP1R‐neurons; *n* = 10, (E) NPY2R‐neurons; *n* = 10; error bars represent SD. Data were analyzed using Student's *t*‐test. *P < 0.05 vs. ligand‐free samples (0 nm). (B, D, F) Ratio of change in neuronal firing frequency with ligand addition, determined by MEA recording. Left panels present phase‐contrast images of neurons seeded on MEA substrates. (B) CCKAR‐neurons; day 27, 0 nm: *n* = 128 electrodes, 100 nm: *n* = 57 electrodes. (D) GLP1R‐neurons; day 27, 0 nm: *n* = 31 electrodes, 10 nm: *n* = 34 electrodes. (F) NPY2R‐neurons; day 27, 0 nm: *n* = 8 electrodes, 100 nm: *n* = 6 electrodes. Error bars represent the SD. Data were analyzed using Student's *t*‐test. *P < 0.05, **P < 0.01 vs. ligand‐free samples (0 nm). See also Fig. S5.

The ligand responsiveness of established neurons was verified using calcium imaging and microelectrode arrays (MEA) recording. The ligand concentration was determined based on the results of agonist studies on CCKAR‐HEK293T cells (Fig. 2A). The results revealed that 100 nm CCK‐33 significantly increased the intracellular Ca^2+^ concentration (Fig. 6A, Fig. S5A). We also observed that CCK‐33 significantly increased the neuronal firing frequency change rate (Fig. 6B) (100 nm vs. 0 nm CCK‐33: *P* = 0.018).

Moreover, 10 nm GLP1 increased the intracellular Ca^2+^ concentration of selected GLP1R‐neurons (Fig. S5B); the ratio of change (Δ*F*_stim_/*F*_0_) in the mean fluorescence intensity before and after ligand addition was significantly increased (Fig. 6C). Moreover, GLP1 increased the change rate of firing frequency in GLP1R‐neurons (Fig. 6D).

Similarly, 100 nm PYY increased intracellular Ca^2+^ concentration in NPY2R‐neurons (Fig. S5C). PYY significantly increased fluorescence intensity and firing frequency in NPY2R‐neurons (Fig. 6E,F). Thus, the iPSC‐derived engineered neurons strongly expressed GI hormone receptors and exhibited robust ligand responsiveness.

**Fig. 7:**
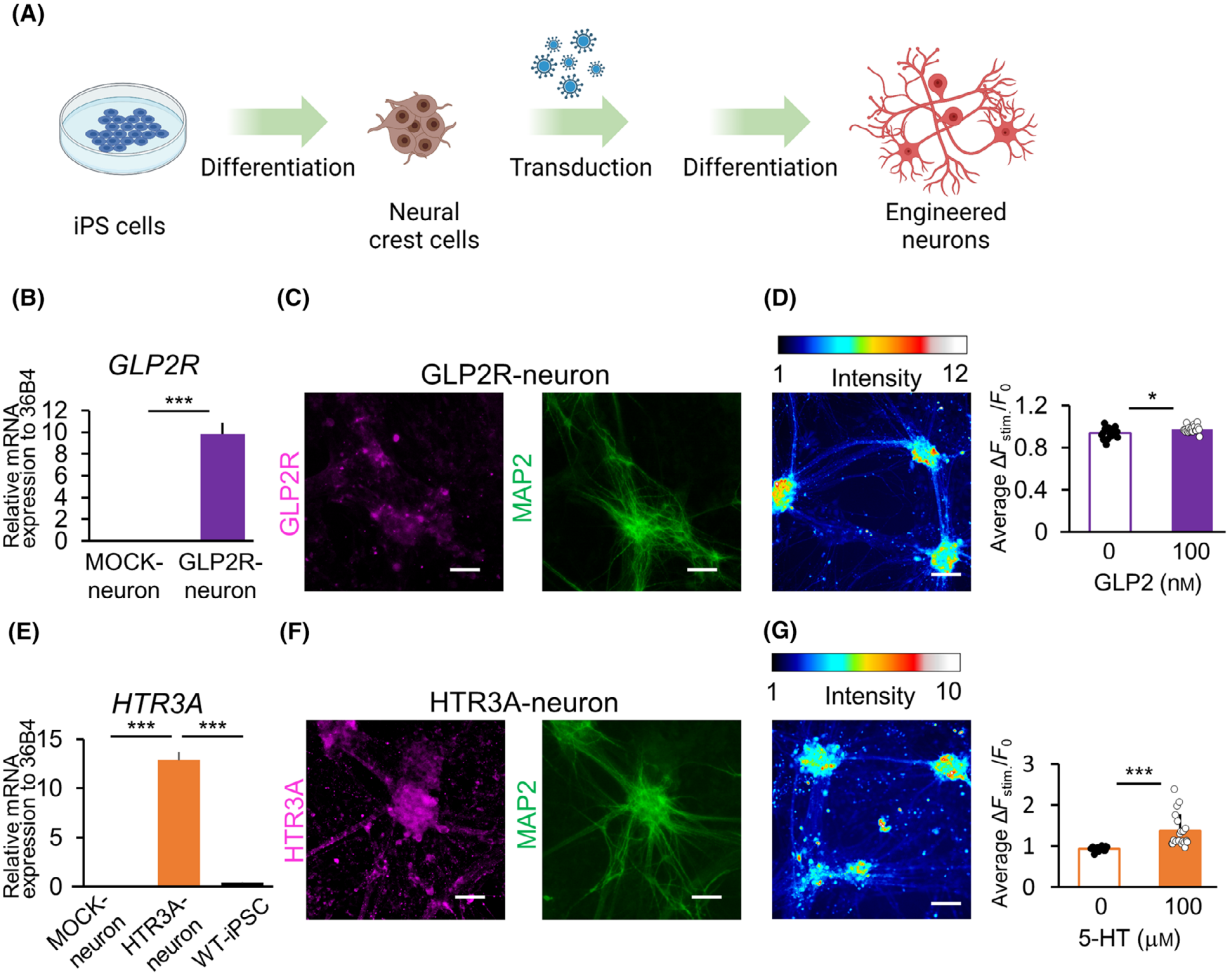
Establishment and verification of the responsiveness of directly transduced GLP2R‐ or HTR3A‐neurons. (A) Schematic diagram of the process of establishing GI hormone receptor‐expressing neurons via gene transduction during differentiation from NCCs to parasympathetic neurons. (B–D) showed GLP2R, (E–G) showed HTR3A results. (B, E) mRNA expression levels of *GLP2R* or *HTR3A* to those of *36B4* (housekeeping gene) in WT‐iPSCs, neurons transduced with MOCK during the differentiation process (MOCK‐neurons), and neurons transduced with GLP2R (GLP2R‐neurons) or HTR3A (HTR3A‐neurons) on day 40 (*n* = 3; error bars represent the SD). (B) Student's *t*‐test, ***P < 0.001 vs. MOCK‐neurons. (E) One‐way ANOVA followed by Tukey–Kramer's *post‐hoc* test, ***P < 0.001 vs. HTR3A‐neurons. (C, F) Immunofluorescence staining of GI hormone receptors on neurons on day 40 (magenta: GI hormone receptors, green: MAP2, Scale bar: 100 μm) (D, G) Ca^2+^ imaging of neurons transduced with GLP2R or HTR3A during differentiation on day 35 in response to ligands. Color bars represent fluorescence intensity. Scale bar: 100 μm. Right panel presents the change ratio (Δ*F*_stim_/*F*_0_) of the average fluorescence intensity after (Δ*F*_stim_) and before ligand addition (*F*_0_). *n* = 20; error bars represent SD. Data were analyzed using Student's *t*‐test. *P < 0.05, ***P < 0.001 vs. ligand‐free samples (0 nm or 0 μm). See also Figs S6 and S7.

As *GLP2R* and *HTR3A* were not expressed in neurons from the established iPSC lines, we tried to generate neurons expressing GI hormone receptors by performing direct gene transfer into neurons during the differentiation process from NCCs to neurons. First, parasympathetic neurons were induced from WT‐iPSCs, and the *GLP2R* or *HTR3A* gene was transduced into cells on day 20 of the neuronal maturation process (Fig. 7A). The neurons exhibited the expression of parasympathetic nerve markers (*PRPH* and *CHAT*) on day 40 (Fig. S6A), indicating that gene transfer might not affect neuronal differentiation. The engineered neurons exhibited higher expression of GLP2R or HTR3A than WT‐neurons (GLP2R‐neurons and HTR3A‐neurons, Fig. 7B,E). MAP2‐positive neurons also expressed GLP2R or HTR3A protein (Fig. 7C,F). Additionally, Ca^2+^ imaging revealed that GLP2R‐neurons responded slightly to 100 nm GLP2, with a 3% percentage change in fluorescence intensity (Fig. 7D, Fig. S7A). Furthermore, HTR3A‐neurons showed increased intracellular Ca^2+^ concentration in response to 100 μm 5‐HT (Fig. 7G, Fig. S7B). Thus, the generated neurons responded to GLP2 and 5‐HT by transducing the genes during neuronal differentiation.

## Discussion

We generated HEK293T cells, iPSC lines, and induced parasympathetic neurons that respond to GI hormones. This work aims to contribute to a better understanding of the interactions between these neurons and GI hormones, and potentially provide insights into the complex brain–gut axis. Previous research identified specific afferent subtypes [29] and efferent nerves [30] that show differential expression of either specific GI hormone receptors or multiple ones. Using the strategy established in the present study, it is possible to develop neurons expressing single or multiple receptor types, accurately reflecting endogenous vagal nerve tracts.

Previous reports indicate that rat nodal ganglion cells respond to various concentrations of hormones, including CCK, GLP1, PYY, and 5‐HT [31, 32, 33, 34]. With similar concentrations to these findings, this study showed that CCK‐33, GLP1, PYY, GLP2, and 5‐HT led to amplified Ca^2+^ levels in CCKAR‐, GLP1R‐, NPY2R‐, GLP2R‐, and HTR3A‐neurons, respectively. These results suggest that the induced neurons might reproduce nerve activities typically mediated by these hormones. However, it is noteworthy that the overexpression of these receptors in our model may not reconstruct natural expression patterns of endogenous receptors. Furthermore, the overexpression of genes can lead to unintended consequences, such as the disruption of intracellular signaling pathways. Consequently, it is crucial to ascertain whether the response of the generated neurons is truly reflective of *in vivo* responses.

CCK and GLP1 are implicated in neuroprotection and neurite outgrowth within the central nervous system (CNS) [35, 36], with GLP1R agonists offering therapeutic avenues for Alzheimer's and Parkinson's disease [37, 38, 39]. The neuroprotective role of GLP1 in diabetic polyneuropathy and the influence of serotonin in cognitive decline post‐COVID‐19 further substantiate the significance of the brain–gut axis [36, 40]. Additionally, the involvement of NPY2R in cardiovascular reflexes opens new research avenues for syncope [41]. Hence, GI hormone receptor‐expressing iPSC‐derived neurons can significantly contribute to understanding these mechanisms and aiding in therapeutic development. Beyond the impact of CCK on gallbladder function and the observed overexpression of CCKAR in specific cancers [42], our cellular model may also inform targeted anticancer therapies.

We specifically focused on GLP2R. Although RNA‐seq validated the ectopic expression of GLP2R in GLP2R‐iPSCs, only a minority of iPSCs and the derived neurons presented GLP2R protein expression. This contrasted with the consistent mRNA and protein expression for GLP2R observed in both GLP2R‐HEK293T cells and directly induced neurons. Notably, GLP2R‐iPSCs displayed aggregated proteins at certain intracellular locations, suggesting potential yet unidentified mechanisms or regulators that might influence GLP2R protein translation or stability in different cells. Although GLP2R expression in rat and mouse nodose nerves is established, there is a debate about the role of GLP2 in the vagus tract [43, 44]. This developed strategy might be instrumental in gaining more clarity to this area.

The role of HTR3A in preserving the undifferentiated state of ESCs and iPSCs is documented [45]. This suggests that its elevated expression may influence iPSC differentiation. Overexpression of HTR3A mRNA and protein was observed in HTR3A‐iPSCs but was absent in the derived HTR3A‐neurons. As they differentiated into parasympathetic nerves, vector‐derived drug resistance was observed without any enforced expression of HTR3A mRNA or protein. This indicates potential mechanisms, such as the sole insertion of the drug‐resistance gene into the genome or unknown factors affecting HTR3A mRNA expression and protein stability. When neurons were directly induced for differentiation, those that stably expressed HTR3A showed responsiveness to 5‐HT, reinforcing the critical role of the timing of gene introduction.

The identity of the parasympathetic neurons created using the current induction method, as well as their representation as vagus nerves, remains a topic of discussion. Validation would necessitate assessing gene expression linked with vagal differentiation, examining neurotransmitter release upon stimulation, and evaluating their potential in modulating brain and peripheral tissue functionalities. The responsiveness of the vagus nerve to a plethora of stimuli, such as bile acids, cytokines, and physical stimuli [46, 47, 48, 49, 50], further underscores its significance. Confirming the identity of these neurons would provide an invaluable model for studying responses to a broad spectrum of exposomes, thereby deepening our understanding of the brain–gut connection. This could potentially pave the way for novel therapeutic strategies targeting various conditions like Parkinson's disease, depression, irritable bowel syndrome, and other GI and psychiatric ailments [51, 52, 53].

Lastly, the established neurons expressing GI hormone receptors can be useful for studying hormone‐related neuronal functions and investigating new compounds that can influence neuronal activity through these receptors. Future endeavors should aim to identify the optimal concentration threshold for each ligand to determine nerve responsiveness to hormones. These bioengineered neurons can potentially provide *in vitro* models, which may reduce reliance on traditional animal experiments. Their use could be broadened to encompass non‐clinical investigations, including those involving natural substances and a variety of chemical compounds.

## Conflict of interest

YSK received research grants from Asahi Quality & Innovations, Ltd. YSK and YT are the inventors of patent JP6593811. The other authors declare no conflict.

### Peer review

The peer review history for this article is available at https://www.webofscience.com/api/gateway/wos/peer‐review/10.1002/2211‐5463.13741.

## Author contributions

YM, YA, YT, and YSK were involved in conceptualization; YA, YT, and YSK were involved in methodology; YA and YT were involved in validation and formal analysis; YA, YM, AY, RY, and YSK were involved in investigation; YA and YSK were involved in data curation, funding acquisition, supervision and writing—original draft preparation; YA, YT, YN, YM, AY, RY, and YSK were involved in writing—review and editing; YA was involved in visualization. All authors have read and agreed to the published version of the manuscript.

## Supporting information


**Fig. S1.** Schematic diagram of the protocol for generating parasympathetic neurons expressing gastrointestinal (GI) hormone receptors.
**Fig. S2.** Confirmation of responsiveness of each receptor using HEK293T cells expressing CCKAR and NPY2R.
**Fig. S3.** Verification of genomic stability and differentiation capacity of engineered iPSCs.
**Fig. S4.** Comparison of transgene expression in HTR3A‐transduced neurons and undifferentiated iPS cells.
**Fig. S5.** Verification of ligand responsiveness of engineered neurons (CCKAR‐neurons, GLP1R‐neurons, and NPY2R‐neurons).
**Fig. S6.** Establishment of GLP2R‐ and HTR3A‐transduced neurons during the differentiation process.
**Fig. S7.** Establishment and responsiveness of GLP2R‐ or HTR3A‐transduced neurons during the differentiation process.
**Table S1.** Names of plasmids and iDs of lentiviral vectors used in this study. mCherry‐transduced cells (MOCK) were used as the control.
**Table S2.** Sequences of primers used for quantitative PCR.

## Data Availability

mRNA sequencing (RNA‐seq) data are deposited in the DDBJ under the accession number DRA015313. RNA‐seq data for NCCs on induction day 13 (negative control) are deposited under the accession number DRA008963. Further information and requests regarding resources and reagents should be directed to and will be fulfilled by the corresponding author, Yasuyuki S Kida (y-kida@aist.go.jp). All unique/stable reagents generated in this study will be made available on reasonable request; however, we may require a payment and/or a completed Materials Transfer Agreement if there is potential for commercial application.
