# Supplementary material for: Functional engineering of human iPSC‐derived parasympathetic neurons enhances responsiveness to gastrointestinal hormones
Source: FEBS Open Bio. 2023 Dec 12;14(1):63–78. doi: 10.1002/2211-5463.13741 (PMC10761937; doi:10.1002/2211-5463.13741)
Supplement: Supplementary file 1 — Fig. S1. Schematic diagram of the protocol for generating parasympathetic neurons expressing gastrointestinal (GI) hormone receptors. Fig. S2. Confirmation of responsiveness of each receptor using HEK293T cells expressing CCKAR and NPY2R. Fig. S3. Verification of genomic stability and differentiation capacity of engineered iPSCs. Fig. S4. Comparison of transgene expression in HTR3A‐transduced neurons and undifferentiated iPS cells. Fig. S5. Verification of ligand responsiveness of engineered neurons (CCKAR‐neurons, GLP1R‐neurons, and NPY2R‐neurons). Fig. S6. Establishment of GLP2R‐ and HTR3A‐transduced neurons during the differentiation process. Fig. S7. Establishment and responsiveness of GLP2R‐ or HTR3A‐transduced neurons during the differentiation process. Table S1. Names of plasmids and iDs of lentiviral vectors used in this study. mCherry‐transduced cells (MOCK) were used as the control. Table S2. Sequences of primers used for quantitative PCR. [file FEB4-14-63-s001.docx]

**Supplemental Information**

**Supplemental figures**

**
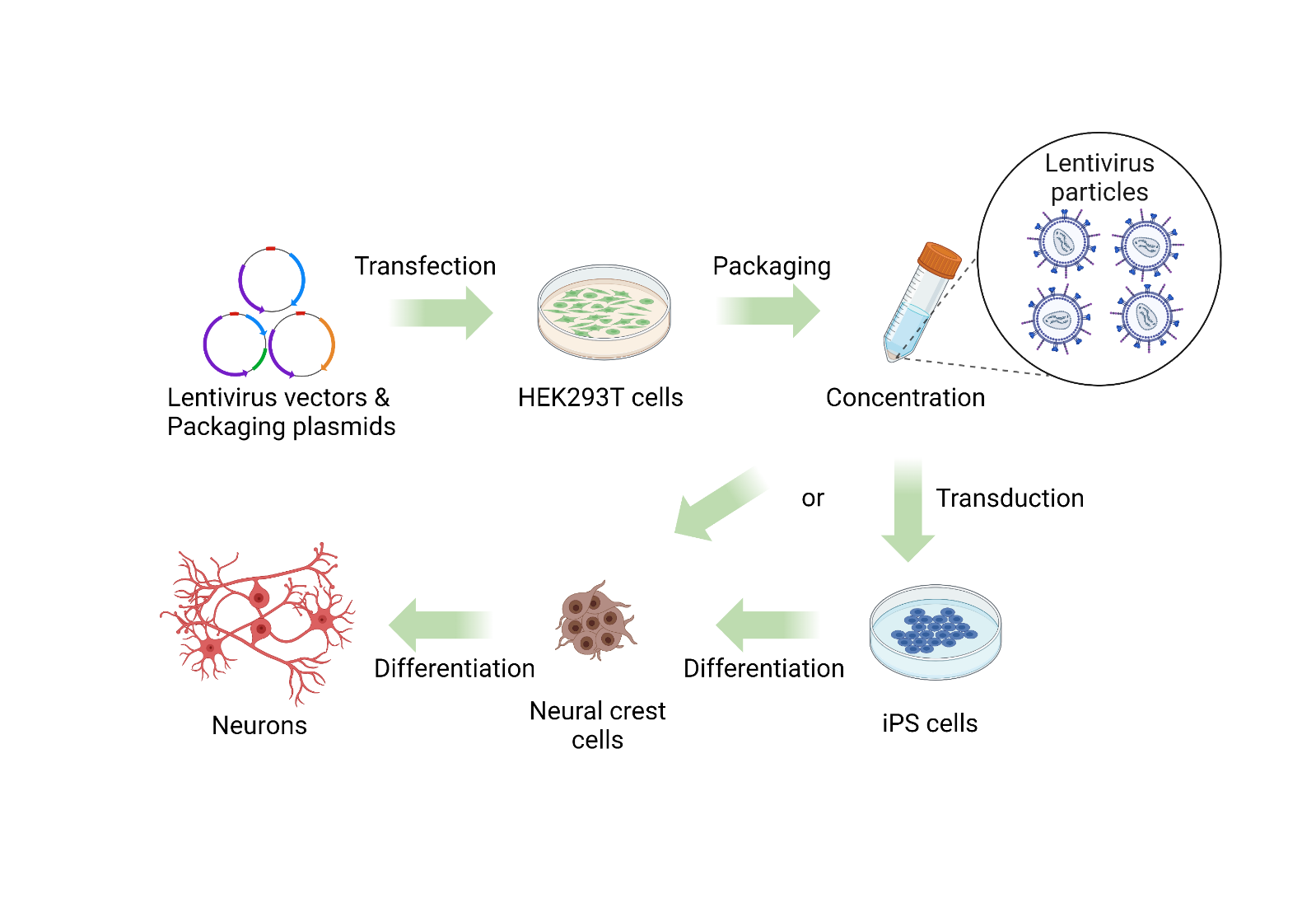
**

**Figure S1.** Schematic diagram of the protocol for generating parasympathetic neurons expressing gastrointestinal (GI) hormone receptors.

**
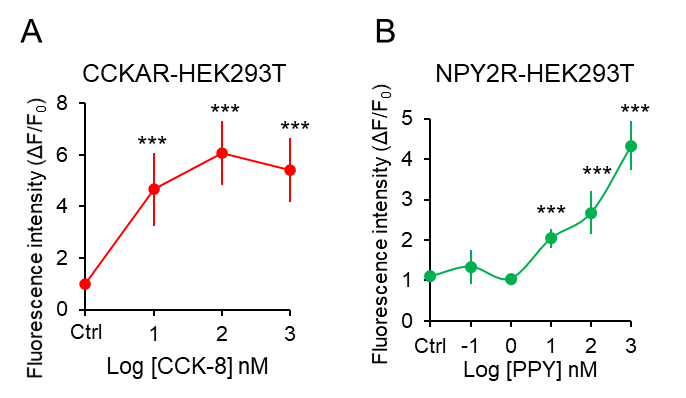
**

**Figure S2.** Confirmation of responsiveness of each receptor using HEK293T cells expressing CCKAR and NPY2R. (**A**) Concentration-response curve of intracellular Ca^2+^ concentration (ΔF/F_0_) of CCKAR-HEK293T cells in response to CCK-8 treatment (*n* = 10; error bars represent SD). (**B**) Concentration-response curve of intracellular Ca^2+^ concentration (ΔF/F_0_) of NPY2R-HEK293T cells in response to PYY (3-36) treatment (*n* = 10; error bars represent SD). Data were analyzed using one-way ANOVA followed by Dunnett’s post-hoc test, *** *P* < 0.001 vs. 0 nM (Control; Ctrl). (**A, B**) ΔF: average fluorescence after ligand addition, F_0_: average fluorescence before ligand addition.

**
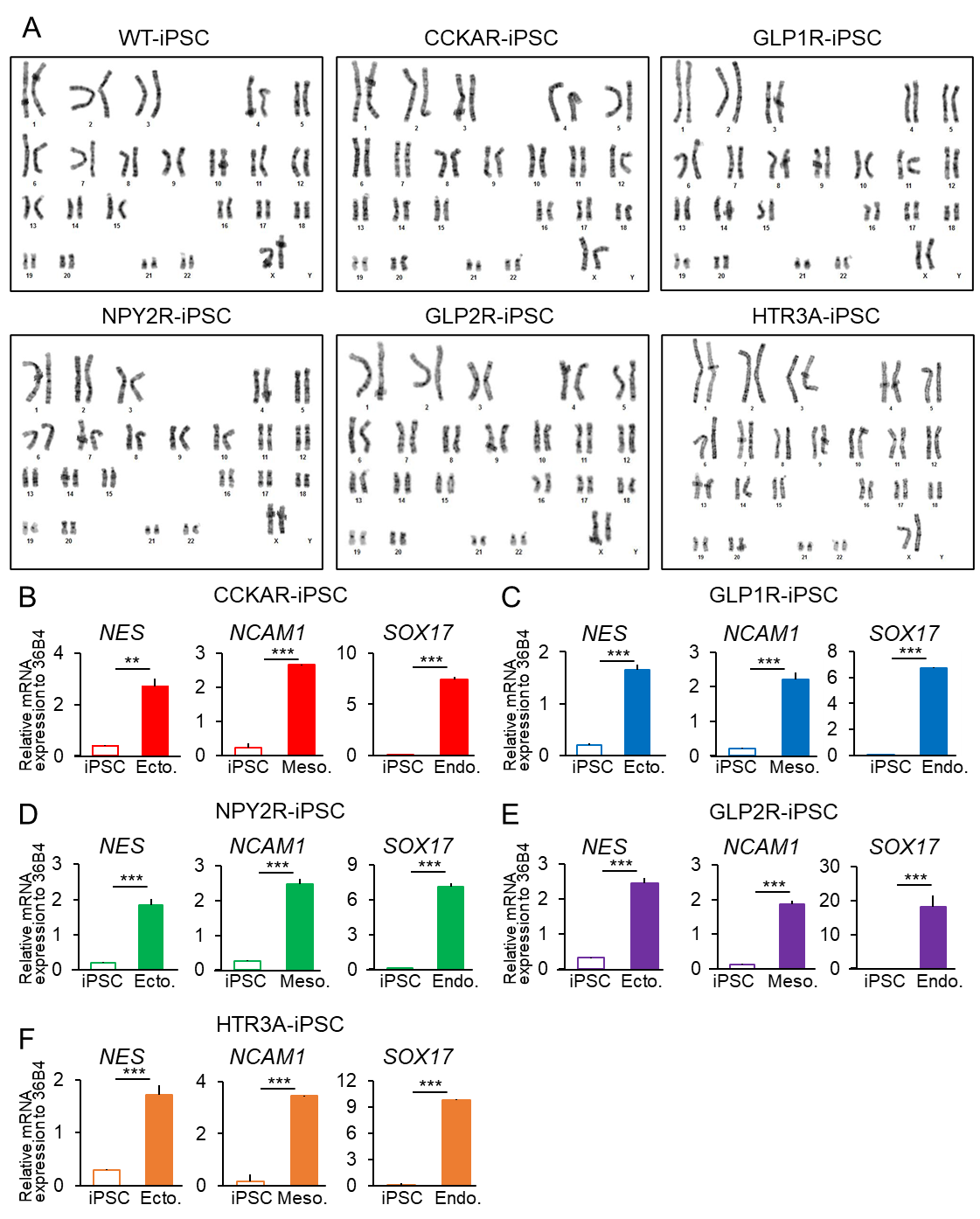
**

**Figure S3.** Verification of genomic stability and differentiation capacity of engineered iPSCs. **(A)** Cytogenetic analysis via G-band staining showing 46 chromosomes, including the sex chromosomes (XX). (**B‒F**) mRNA expression levels of nestin (*NES*)*,* *NCAM1*, and *SOX17* compared to that of *36B4* (housekeeping gene) in each engineered iPSC line differentiated into ectoderm (Ecto.), mesoderm (Meso.), and endoderm (Endo.). Receptor-transduced iPSCs before differentiation were used as controls. Data were analyzed using Student’s *t*-test. *n* = 3; error bars represent SD. ** *P* < 0.01, *** *P* < 0.001 vs. undifferentiated cells (iPSCs). CCKAR-iPSC, Ecto., Meso., and Endo. were used in (**B**). GLP1R-iPSC, Ecto., Meso., and Endo. were used in (**C**). NPY2R-iPSCs, Ecto., Meso., and Endo. were used in **(D)**. GLP2R-iPSCs, Ecto., Meso., and Endo. were used in (**E**). HTR3A-iPSCs, Ecto., Meso., and Endo. were used in (**F**).


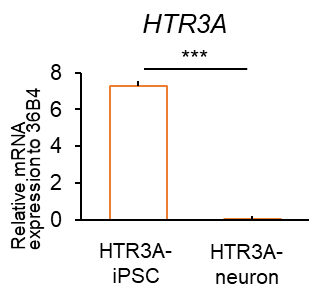


**Figure S4.** Comparison of transgene expression in HTR3A-transduced neurons and undifferentiated iPS cells. mRNA expression levels of *HTR3A* compared to that of *36B4* in HTR3A-transduced iPSC and neurons on day 40. Data were analyzed using Student’s *t*-test. *n* = 3. Error bars represent SD. *** *P* < 0.001 vs. HTR3A-iPSCs.

**
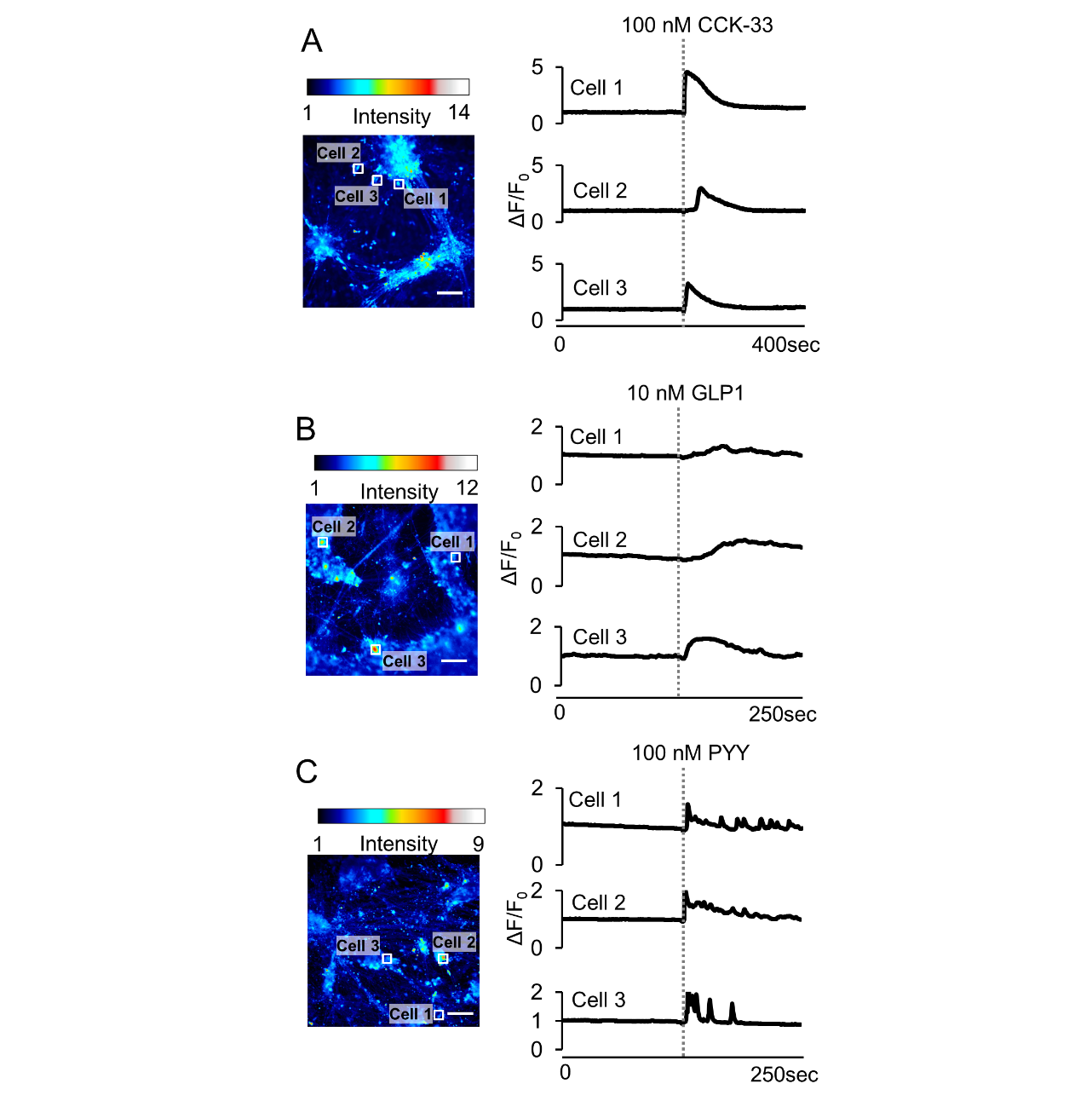
**

**Figure S5.** Verification of ligand responsiveness of engineered neurons (CCKAR-neurons, GLP1R-neurons, and NPY2R-neurons). (**A**) Responsiveness of CCKAR-neurons to 100 nM CCK. (**B**) Responsiveness of GLP1R-neurons to 10 nM GLP1. (**C**) Responsiveness of NPY2R-neurons to 100 nM PYY. (**A‒C**) Ca^2+^ imaging of engineered neurons on day 35 in response to the ligands. Color bars represent fluorescence intensity. Right panels show typical traces of calcium transients (ΔF/F_0_) in the three cells in the fluorescence image. F_0_: average fluorescence intensity before ligand addition (**A** 0–200 s, **B** and **C** 0–125 s). Scale bar: 100 μm.

**
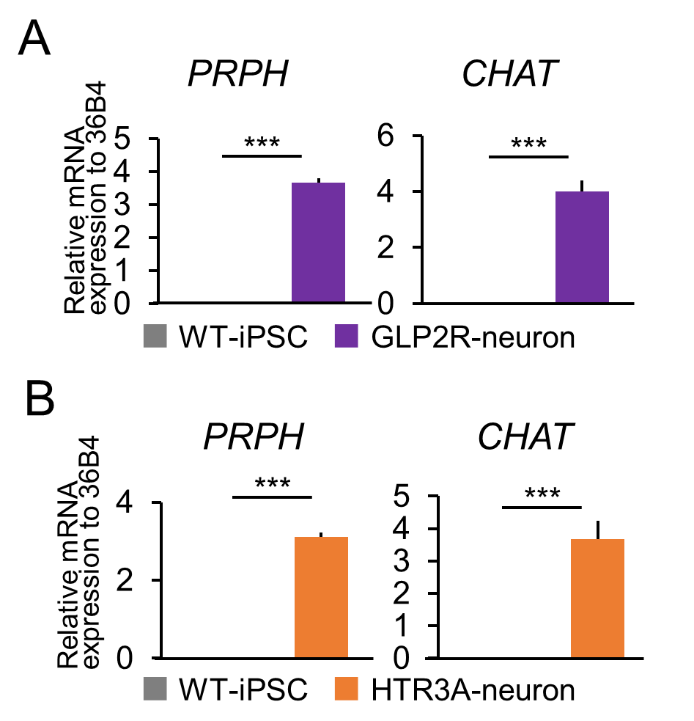
**

**Figure S6.** Establishment of GLP2R- and HTR3A-transduced neurons during the differentiation process. (**A, B**) mRNA expression levels of peripheral nerve marker genes (peripherin (*PRPH*) and *CHAT*) compared to that of *36B4* in WT-iPSCs and neurons transduced with *GLP2R* or *HTR3A* during differentiation (generating GLP2R-neuron and HTR3A-neuron, respectively) on day 40. Data were analyzed using Student’s *t*-test. *n* = 3. Error bars represent SD. *** *P* < 0.001 vs. WT-iPSCs.

**
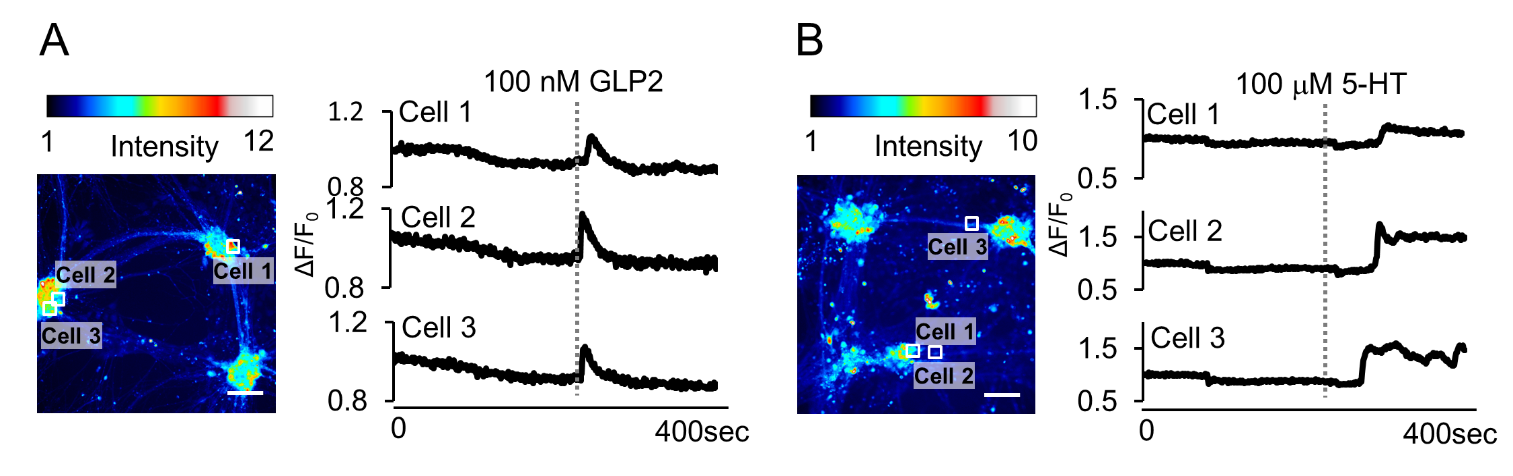
**

**Figure S7.** Establishment and responsiveness of GLP2R- or HTR3A-transduced neurons during the differentiation process. (**A**) Responsiveness of neurons transduced with GLP2R during differentiation (GLP2R-neurons) to 100 nM GLP2. (**B**) Responsiveness of neurons transduced with HTR3A during differentiation (HTR3A-neurons) to 100 μM 5-HT. (**A, B**) Ca^2+^ imaging of engineered neurons on day 35 in response to the ligands. Color bars represent fluorescence intensity. Right panels present typical traces of calcium transients (ΔF/F_0_) in the three cells in the fluorescence image. F_0_: average fluorescence intensity before ligand addition (0–200 s). Scale bar: 100 μm.

**Supplemental tables**

**Table S1.** Names of plasmids and iDs of lentiviral vectors used in this study. mCherry-transduced cells (MOCK) were used as the control. The plasmid iDs were obtained from Vector Builder Inc. (<https://www.vectorbuilder.jp/>).

| Gene symbol | NCBI number | Plasmid name | Plasmid ID |
| --- | --- | --- | --- |
| mCherry | - | pLV[Exp]-Puro-EF1A>mCherry | VB900084-0158zxv |
| CCKAR (Cholecystokinin A receptor) | NM_000730.3 | pLV[Exp]-Puro-EF1A>hCCKAR | VB900123-1385gxm |
| GLP1R (Glucagon-like peptide-1 receptor) | NM_002062.5 | pLV[Exp]-Puro- EF1A>hGLP1R | VB900123-1388xwv |
| GLP2R (Glucagon-like peptide-2 receptor) | NM_004246.3 | pLV[Exp]-Puro-EF1A>hGLP2R | VB210830-1115xup |
| HTR3A (5-hydroxytryptamine receptor 3A) | NM_213621.4 | pLV[Exp]-Puro-EF1A>hHTR3A | VB210823-1277qqk |
| NPY2R (Neuropeptide Y Receptor Y2) | NM_000910.4 | pLV[Exp]-Puro-EF1A>hNPY2R | VB900123-1387bag |

**Table S2.** Sequences of primers used for quantitative PCR.

| Gene symbol | Forward (5'-3') | Reverse (5'-3') |
| --- | --- | --- |
| *36B4* | AGATGCAGCAGATCCGCA | GTTCTTGCCCATCAGCACC |
| *CCKAR* | AGGATTTCATCTTCGGGAGCG | CGGGACTGTAAGGGTTTGC |
| *CHAT* | GCCTTCTACAGGCTCCATCG | GGAGTGGCCGATCTGATGTT |
| *GLP1R* | CTACGCACTCTCCTTCTCTGCT | CGGACAATGCTCGCAGGATGAA |
| *GLP2R* | GGAAGTGGGCTCAGTACAAA | GTCCCGTTACAAAATATGCCAG |
| *HTR3A* | CATCTTCATTGTGCGGCTGGTG | AGTCATCAGTCTTGGTGGCTTGG |
| *NANOG* | CCAACATCCTGAACCTCAGC | GCTATTCTTCGGCCAGTTG |
| *NCAM1* | CCGTCATCCTGCTTGATCAG | GAGTTCAAGACGCAGCCA |
| *NESTIN* | CGCTCAGGTCCTGGAAGGTCG | AAGTCTTGGAGCCACCGCCA |
| *NPY2R* | CATCTTGCTTGGGGTAATTGGC | AGAGTGAACGGTAGACACAGAG |
| *PHOX2B* | GCTGGCCCTGAAGATCGAC | TCAGACTTTTTGCCCGAGGAG |
| *PRPH* | GCCTGGAACTAGAGCGCAAG | CCTCGCACGTTAGACTCTGG |
| *SOX10* | CCTCACAGATCGCCTACACC | CATATAGGAGAAGGCCGAGTAGA |
| *SOX17* | GGCGCAGCAGAATCCAGA | CCACGACTTGCCCAGCAT |
